# Modeling Down Syndrome Myeloid Leukemia by Sequential Introduction of *GATA1* and *STAG2* Mutations in Induced Pluripotent Stem Cells with Trisomy 21

**DOI:** 10.3390/cells11040628

**Published:** 2022-02-11

**Authors:** Sonali P. Barwe, Aimy Sebastian, Ishnoor Sidhu, Edward Anders Kolb, Anilkumar Gopalakrishnapillai

**Affiliations:** 1 Nemours Centers for Childhood Cancer Research & Cancer and Blood Disorders, Nemours Children’s Health, Wilmington, DE 19803, USA; sbarwe@nemours.org (S.P.B.); ishnoor.sidhu@nemours.org (I.S.); eakolb@nemours.org (E.A.K.); 2 Department of Biological Sciences, University of Delaware, Newark, DE 19711, USA; 3 Physical and Life Sciences Directorate, Lawrence Livermore National Laboratory, Livermore, CA 94550, USA; sebastian4@llnl.gov

**Keywords:** Down syndrome, iPSC, CRISPR/Cas9, leukemia, GATA1s, *STAG2*

## Abstract

Children with Down syndrome (DS) have a high risk for acute myeloid leukemia (DS-ML). Genomic characterization of DS-ML blasts showed the presence of unique mutations in *GATA1*, an essential hematopoietic transcription factor, leading to the production of a truncated from of GATA1 (GATA1s). GATA1s, together with trisomy 21, is sufficient to develop a pre-leukemic condition called transient abnormal myelopoiesis (TAM). Approximately 30% of these cases progress into DS-ML by acquisition of additional somatic mutations in a stepwise manner. We previously developed a model for TAM by introducing disease-specific *GATA1* mutation in trisomy 21-induced pluripotent stem cells (iPSCs), leading to the production of N-terminally truncated short form of GATA1 (GATA1s). In this model, we used CRISPR/Cas9 to introduce a co-operating mutation in *STAG2*, a member of the cohesin complex recurrently mutated in DS-ML but not in TAM. Hematopoietic differentiation of *GATA1* *STAG2* double-mutant iPSC lines confirmed GATA1s expression and the loss of functional STAG2 protein, leading to enhanced production of immature megakaryocytic population compared to *GATA1* mutant alone. Megakaryocyte-specific lineage expansion of the double-mutant HSPCs exhibited close resemblance to the DS-ML immunophenotype. Transcriptome analysis showed that *GATA1* mutation resulted in downregulation of megakaryocytic and erythrocytic differentiation pathways and interferon α/β signaling, along with an upregulation of pathways promoting myeloid differentiation such as toll-like receptor cascade. The co-occurrence of *STAG2* knockout partially reverted the expression of genes involved in myeloid differentiation, likely leading to enhanced self-renewal and promoting leukemogenesis. In conclusion, we developed a DS-ML model via hematopoietic differentiation of gene-targeted iPSCs bearing trisomy 21.

## 1. Introduction

Down syndrome (DS), characterized by trisomy 21, is known to be a leukemia-predisposing syndrome [1]. The dosage imbalance in the hematopoiesis governing genes (*RUNX1*, *DYRK1A*) located on chromosome 21 [2,3] is considered to be the likely cause of the 500-fold higher incidence of myeloid leukemia in young children with DS (DS-ML) [4]. Early acquisition of somatic mutations in *GATA1*, resulting in the production of N-terminally truncated short form of GATA1 protein (GATA1s) [5,6,7], is linked to the induction of transient abnormal myelopoiesis (TAM) seen in 1 of 10 DS infants [8,9]. Thus, trisomy 21 and *GATA1* mutation together induce this pre-leukemic condition characterized by increased population of megakaryoblasts in the peripheral blood [10]. Although TAM in most infants is self-resolved, approximately one-third of these representing 1–2% of DS children develop DS-ML prior to age 5 [9]. This process is believed to be promoted by the acquisition of additional somatic mutations in genes encoding three major classes of proteins—cohesin complex components, epigenetic regulators, and signaling molecules [10,11,12].

Comprehensive genomic analyses of DS-ML and TAM blasts showed the presence of mutations in one or other cohesin complex components in 53% DS-ML patients, while no such mutations were observed in TAM patients [12]. The mutations in cohesin complex components were mutually exclusive, indicating that they constituted driver mutations. The cohesin complex functions to hold sister chromatids together until they are segregated. *STAG2* (stromal antigen 2), a redundant subunit of cohesin complex, is the most frequently mutated cohesin complex component in several types of cancers [13]. In addition to its redundant role in sister chromatid assembly, *STAG2* is a transcriptional coactivator shown to regulate stem cell expansion and differentiation [14].

We recently reported an in vitro disease model by customizing trisomic induced pluripotent stem cells (iPSCs) using precise *GATA1* gene editing to recapitulate the characteristics of TAM [15]. We utilized this model system and CRISPR (clustered regularly interspaced short palindromic repeats)/Cas9 methodology for the introduction of loss of function mutations in *STAG2*, representing the third genetic hit (besides trisomy 21 and GATA1s) to develop DS-ML. Hematopoietic differentiation of two distinct trisomic iPSC lines bearing *GATA1* and *STAG2* mutation showed that GATA1s and loss of *STAG2* protein in trisomy 21 background co-operatively increased the abundance of the megakaryoid population and promoted expression of DS-ML markers. Thus, we developed an iPSC system to model the stepwise mutagenesis in DS-ML leukemogenesis using CRISPR/Cas9-mediated gene targeting.

## 2. Materials and Methods

### 2.1. iPSC Lines and Culture

Isogenic iPSC lines with trisomy 21 (T21-1) and disomy 21 (D21-1) (described in [15] as T21 and D21, respectively) were obtained from RUCDR Infinite Biologics, Rutgers University, NIH Center for Regenerative Medicine [16]. Trisomic DS2-iPS10 iPSC line (T21-2) (described in [15] as H) was gifted by Prof. George Daley, Children’s Hospital, Harvard University, Boston, MA [17]. 

iPSCs were cultured in complete mTESR1 (StemCell Technologies, Ontario, Canada) on Matrigel (Corning, Tewksbury, MA, USA)-coated plates. Subculturing was performed by dissociating iPSC colonies with cell dissociation agent (StemCell Technologies) for 3 min followed by scraping the colonies into the mTESR1 media. Whenever colonies were revived from liquid nitrogen, mTESR1 was supplemented with 10 μM Rho Kinase inhibitor Y27632 (Cayman, Ann Arbor, MI, USA) overnight before continuing the culture in fresh mTER1 medium. Cells were tested for their pluripotency by determining the expression of pluripotency markers TRA-1-60 and SSEA4 (BioLegend, San Diego, CA, USA) by flow cytometry. Bioauthentication was performed to confirm the ploidy and integrity of the iPSC lines using the AmpFLSTR Identifiler PCR Amplification kit (ThermoFisher Scientific, Waltham, MA, USA).

### 2.2. CRISPR Design and Cloning

CRISPR guide sequence for *STAG2* was designed using algorithm on http://crispor.tefor.net/, accessed on 19 August 2020 [18]. Alt-R CRISPR-Cas9 sgRNA with single RNA molecule comprising crRNA and tracrRNA was complexed with Alt-R Cas9-GFP nuclease (both obtained from IDT, Coralville, IA, USA) to form RNP complex, which was introduced into iPSCs (0.75 × 105 cells) using a 4D nucleofector system and P3 Primary Cell 4D-Nucleotransfector X Kit L (Lonza; Basel, Switzerland). Transfected cells were cultured in 12-well plates. Two days after transfection, single-cell suspension was generated using accutase, and 10,000 cells were plated in each well of a 6-well plate. Media were changed every day until individual colonies were visible. Individual colonies were expanded for cryopreservation and genomic DNA isolation using a MicroDNA kit (Qiagen, Germantown, MD, USA). Genomic DNA used as a template for PCR using primers flanking the guide sequence. PCR products were Sanger sequenced, and the sequence was analyzed using a free web-based software tool (https://ice.synthego.com, accessed on 24 January 2022). Clones showing desired mutation were further expanded and STAG2 mutation was re-confirmed by Sanger sequencing and immunoblotting.

### 2.3. Hematopoietic Differentiation and Lineage Expansion

For hematopoietic differentiation of iPSC colonies with disease-specific mutation in *STAG2* and *GATA1*, we used a protocol described previously [15]. Briefly, 70–80 uniform-sized colonies were plated on Matrigel-coated 6-well plates. The next day, we added Media A (STEMdiff Hematopoietic kit, StemCell Technologies), and subsequent media changes were performed following the manufacturer’s instructions. Cells collected on day 12 and filtered using sterile cell strainer 100 μm nylon mesh (ThermoFisher Scientific) were analyzed for hematopoietic progenitor cells (HSPCs) by flow cytometry as described below.

For the megakaryocytic lineage expansion, the hematopoietic differentiation was stopped on day 10, the floating cells were collected, and 100,000 cells were resuspended in megakaryocytic lineage expansion media (StemCell Technologies) and continued in culture in a 96-well plate. Media was changed as needed, and on day 5, cells were collected for determination of lineage-specific markers by flow cytometry.

### 2.4. Immunoblot Analysis

Automated immunoblot analysis was performed using Wes system (ProteinSimple, San Jose, CA, USA) according to the manufacturer’s instructions using a 12–230 kDa Separation Module (SM-W001) and the Anti-Rabbit Detection Module (DM-001). iPSCs or HSPCs were lysed in Minute Total Protein Extraction kit (Invent Biotechnologies, Plymouth, MN, USA), sonicated, and clarified by centrifugation at 16,000× *g* for 15 min. Supernatant was collected and protein equivalent to 50,000 cells was loaded per capillary. GATA1, STAG2, pSTAT-1, RIG-I, MDA5, and BCL2 antibodies were purchased from Cell Signaling Technology (Danvers, MA, USA). Normalization to total protein was performed using the Total Protein Detection Module in Wes (DM-TP010).

### 2.5. Flow Cytometry

Cells (25,000) resuspended in staining solution (phosphate-buffered saline (PBS) containing 1% FBS) were stained for 15 min in the dark using appropriate antibodies for the detection of specific cell populations. Brilliant Violet 421-conjugated CD71, FITC-conjugated CD235ab, Brilliant Violet 785-conjugated CD34, APC-conjugated CD41, PE-conjugated CD18, and Brilliant Violet 605-conjugated CD45 were used for analysis of erythroid, megakaryoid, and myeloid population. The total of percent erythroid population (CD71+ CD235ab+), myeloid population (CD45+ CD18+), and megakaryoid population (calculated as the percentage of CD34+ CD41+ cells of total CD41+ cell population) was set at 100.

For analysis of cells generated by megakaryocytic lineage expansion, cells were stained with Pacific blue-conjugated CD56, Brilliant Violet-conjugated 421, Brilliant Violet-conjugated 785, FITC-conjugated CD99, PE-conjugated CD41, and Brilliant Violet 650-conjugated CD42b. Cells were also stained with 1:1000 Hoechst 33,342 for ploidy determination. Cells were washed once with staining solution, centrifuged at 500× *g* for 5 min, and resuspended in 100 μL of staining solution. Samples were acquired on Novocyte 3000 or Quanteon flow cytometers (Agilent Technologies, Palo Alto, CA, USA). Positive events were determined on the basis of respective isotype control antibodies for each fluorophore. Fluorescence compensation was performed on the NovoExpress software using unstained and single antibody-stained cells.

### 2.6. Colony-Forming Unit Assay

To determine the multi lineage-potential, we cultured 1000 HSPCs collected on the 10th day after hematopoietic differentiation in MethoCultTM SF H4636 (StemCell Technologies) for 12 additional days. The three different colonies—CFU-GEMM (colony-forming unit granulocyte erythroid macrophage megakaryocyte), CFU-GM (colony-forming unit granulocyte-macrophage), and BFU-E (burst-forming unit erythroid) were identified and counted using the EVOS M5000 imaging system.

### 2.7. RNA Sequencing and Data Analysis

Total RNA was isolated using Qiagen RNeasy Plus Micro kit following the manufacturer’s protocol. RNA library preparations, sequencing reactions, and initial bioinformatics analysis were conducted at GENEWIZ, LLC. (South Plainfield, NJ, USA). RNA sequencing libraries were prepared using the NEBNext Ultra RNA Library Prep Kit for Illumina following the manufacturer’s instructions (NEB, Ipswich, MA, USA). Briefly, mRNAs were first enriched with Oligo(dT) beads. Enriched mRNAs were fragmented for 15 min at 94 °C. First-strand and second-strand cDNAs were subsequently synthesized. cDNA fragments were end-repaired and adenylated at 3′ ends, and universal adapters were ligated to cDNA fragments, followed by index addition and library enrichment by limited-cycle PCR. The sequencing libraries were validated on the Agilent TapeStation (Agilent Technologies) and quantified by using a Qubit 2.0 Fluorometer (Invitrogen, Carlsbad, CA, USA) as well as by quantitative PCR (KAPA Biosystems, Wilmington, MA, USA). The sequencing libraries were multiplexed and clustered on 6 lanes of a flowcell. After clustering, the flowcell was loaded on the Illumina HiSeq instrument according to the manufacturer’s instructions. The samples were sequenced using a 2 × 150 Paired End (PE) configuration. Image analysis and base calling were conducted by the HiSeq Control Software (HCS). Raw sequence data (.bcl files) generated from Illumina HiSeq was converted into fastq files and de-multiplexed using Illumina’s bcl2fastq 2.17 software. One mismatch was allowed for index sequence identification.

RNA-seq reads were aligned to human reference genome hg38 with STAR [19]. After read alignment, the number of read counts per gene locus was summarized with ‘featureCounts’ [20], using hg38 gene annotation. Prior to statistical analysis, genes with low expression were filtered. The sequencing data were normalized using RUVseq [21] to correct for batch effects and other unwanted variations. Differentially expressed genes were identified using edgeR [22]. Genes with false discovery rate adjusted *p*-value (FDR) less than 0.05 and log2 fold change greater than 0.5 were considered as significantly differentially expressed genes unless otherwise specified. Enriched (FDR less than 0.05) ‘biological processes’ and ‘pathways’ associated with differentially expressed genes were identified using and EnrichR [23]. Gene set enrichment analysis was performed using WebGestalt [24]. Heatmaps and gene set enrichment plots were generated using custom R scripts.

### 2.8. Statistical Analysis

*p*-values to determine the statistical significance of the differences in percent cell population between a pair of iPSC lines were calculated by two-tailed Student’s *t*-test with unequal variance.

## 3. Results

### 3.1. GATA1 and STAG2 Knockout in Trisomic iPSCs by CRISPR/Cas9 Mutagenesis

**Figure 1:**
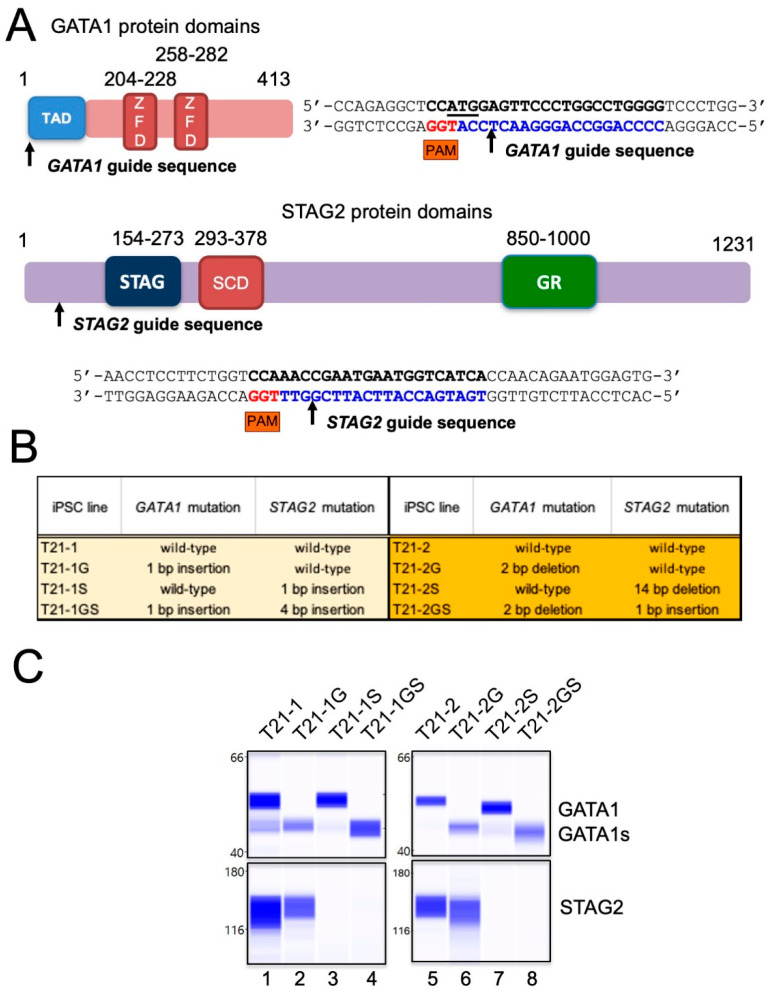
(**A**) Diagrammatic representation showing GATA1 protein domain organization containing two zinc finger domains (ZFD) and a N-terminal transcription activation domain (TAD). Arrow indicates the position of the cut site within the CRISPR guide sequence located in exon 1. Diagrammatic representation showing STAG2 protein domain organization containing STAG domain, stromalin conservative domain (SCD), and glutamine-rich (GR) domain. Arrow indicates the position of the cut site within the CRISPR guide sequence located in exon 5. (**B**) List of *GATA1* and/or *STAG2* mutant iPSC lines generated for the study. Isogenic lines are highlighted by the same color. (**C**) Immunoblots using the automated Western blotting system Wes in band view showing the GATA1, GATA1s, and STAG2. Total protein quantitation was performed to ensure equal loading.

We previously showed that the introduction of patient-specific *GATA1* mutation in trisomic iPSCs leads to the production of a short form of GATA1 (GATA1s) in HSPCs and is sufficient to recapitulate the characteristics of TAM [15]. Trisomy 21 and GATA1s represent the two initial steps in DS-ML leukemogenesis. We used this system for the introduction of additional co-operating mutation in *STAG2* in order to model the DS-ML leukemogenesis. Because an overwhelming majority of *STAG2* mutations observed in DS-ML were nonsense, frameshift, or splice-site alteration resulting in loss of protein function [12], we designed a CRISPR guide sequence located within exon 5 with cut site at the 68th amino acid (Figure 1A). By CRISPR/Cas9 mutagenesis, we introduced *STAG2* mutation in two independent trisomic iPSC lines T21-1 and T21-2, with either wildtype or mutated *GATA1* described previously [15].

Sequence analysis of the genomic DNA region flanking the guide sequence identified clones with either a 1 or 4 bp monoallelic insertion in iPSC line T21-1 derived from the fibroblasts of a female DS individual. The T21 trisomic iPSC line T21-2 derived from a male DS individual displayed a hemizygous deletion of 14 bp or 1 bp insertion, because *STAG2*, like *GATA1*, is located on the X chromosome and there is a single gene copy in these cells. All these mutations resulted in reading frameshift and the introduction of a premature termination codon (Figure 1B). Immunoblot analysis demonstrated the absence of STAG2 protein in CRISPR/Cas9 mutated lines, as well as the exclusive expression of GATA1s in *GATA1* mutant HSPCs (Figure 1C).

### 3.2. Effect of STAG2 Knockout in the Presence or Absence of GATA1 Mutation on Erythroid Differentiation

**Figure 2:**
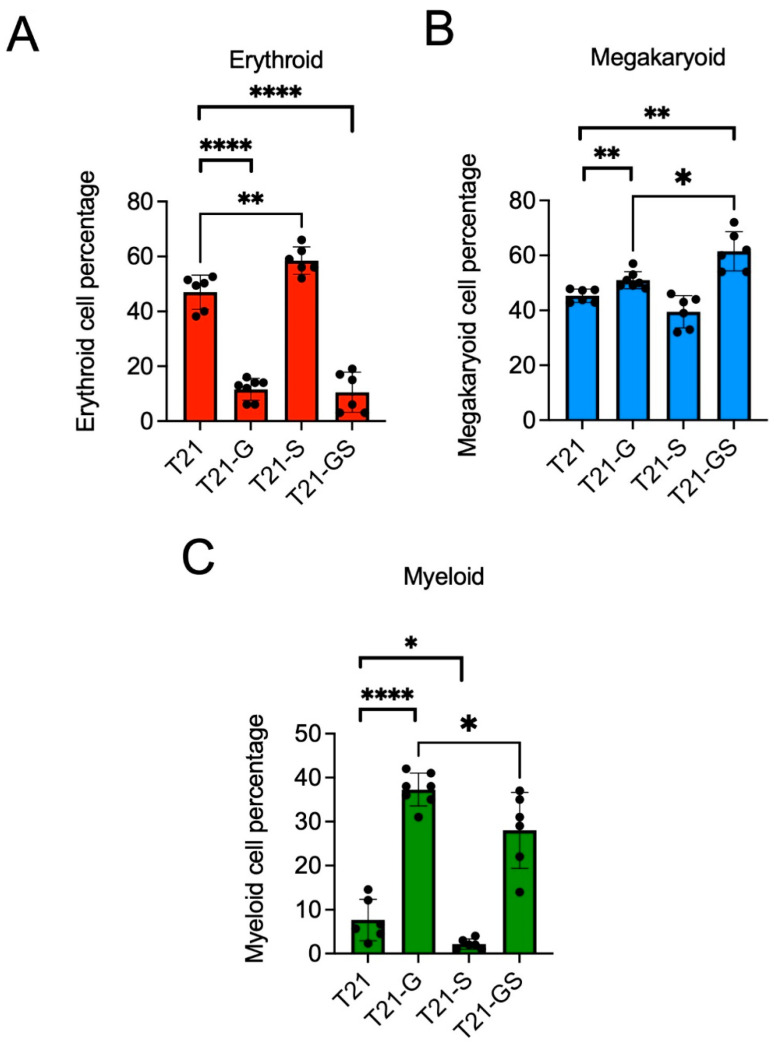
Graphs show the percentage of erythroid (**A**), megakaryoid (**B**), and myeloid (**C**) populations in HSPCs generated from two distinct iPSC lines with or without *GATA1* and/or *STAG2* mutation. Data combined from both iPSC lines performed in 3–4 independent experiments per iPSC line is plotted. Error bars indicate SD of the mean. Asterisk indicates statistical significance in indicated pair of iPSC lines, * *p* < 0.05, ** *p* < 0.01, **** *p* < 0.0001. Data from each iPSC line are presented in Figure S1.

iPSC lines harboring *GATA1* and/or *STAG2* mutations were haematopoietically differentiated to study the multi-lineage hematopoietic differentiation potential. On day 12 after differentiation, HSPCs were collected and used for multi-dimensional flow cytometry analysis. The percentage of erythroid population characterized by CD71 and CD235 positivity was reduced from 47.0 ± 6.3% in trisomic lines with wildtype *GATA1* to 11.6 ± 3.9% in trisomic lines with *GATA1* mutation (Figure 2A, *p* < 0.0001; Figure S1), as we and others have shown previously [15,25]. 

Unlike *GATA1* mutation, *STAG2* knockout increased erythroid population by 3.5% and 11.5% compared to iPSC lines with wildtype *GATA1* (*p* = 0.006). This induction of immature erythroid population by *STAG2* knockout was reported previously in CD34+ cells with loss of *STAG2* [26]. Although the erythroid population in the double-mutant HSPCs was significantly reduced compared to unmutated trisomic HSPCs (*p* < 0.0001), there was no significant difference between the erythroid population in *GATA1* mutant HSPCs compared to the double-mutant HSPCs (*p* = 0.758). While GATA1s caused suppression and *STAG2* knockout resulted in a modest expansion of the erythroid population, the double mutant mirrored the effect of GATA1s.

### 3.3. Effect of STAG2 Knockout in the Presence or Absence of GATA1 Mutation on Megakaryoid Differentiation

Megakaryoid population was analyzed as the percentage of immature megakaryoblasts (CD34+ CD41+) within the total CD41+ population as described previously [15]. A statistically significant increase in the percentage of immature megakaryoblasts was observed in GATA1s expressing trisomy 21 HSPCs compared to trisomy 21 HSPCs with wildtype *GATA1* (Figure 2B, 5.7% increase, *p* = 0.004), consistent with our previous study [15]. There was no statistically significant difference in the megakaryoid population in *STAG2* knockout HSPCs compared to wildtype HSPCs (*p* = 0.061), indicating that *STAG2* knockout did not have significant impact on megakaryoid population. Of note, the percentage of megakaryoid population was significantly higher in *GATA1 STAG2* mutant HSPCs (61.3 ± 7.1%) compared to wildtype HSPCs (45.3 ± 2.4%, *p* = 0.002) or *GATA1* mutant (51.1 ± 3.0%, *p* = 0.014) HSPCs. These data show that the knockout of *STAG2* further stimulated the GATA1s-mediated rise in megakaryoid population, indicative of a co-operative role of *GATA1* and *STAG2* mutation in promoting the percentage of megakaryoid population. 

### 3.4. Effect of STAG2 Knockout in the Presence or Absence of GATA1 Mutation on Myeloid Differentiation

The myeloid population defined by the presence of CD18 and CD45 markers was greatly increased in *GATA1* mutant HSPCs (38.4 ± 3.8%) compared to wildtype (7.7 ± 4.7%) (Figure 2C, *p* < 0.0001), in agreement with our prior data [15]. In contrast with the effect of *GATA1* mutation, *STAG2* knockout reduced myeloid population by 5.7% compared to the wildtype HSPCs (*p* = 0.035). The co-occurrence of *GATA1* and *STAG2* mutation resulted in a 10.4% (*p* = 0.046) reduction in myeloid population compared to *GATA1* mutant. Taken together, these data show that *GATA1* and *STAG2* mutations exhibit contrasting effects on myeloid population percentage, and differentiation of iPSC lines harboring both mutations reduced myeloid cell percentage in comparison with *GATA1* mutant iPSC lines.

### 3.5. Effect of GATA1 and STAG2 Mutation on Megakaryocyte Maturation

**Figure 3:**
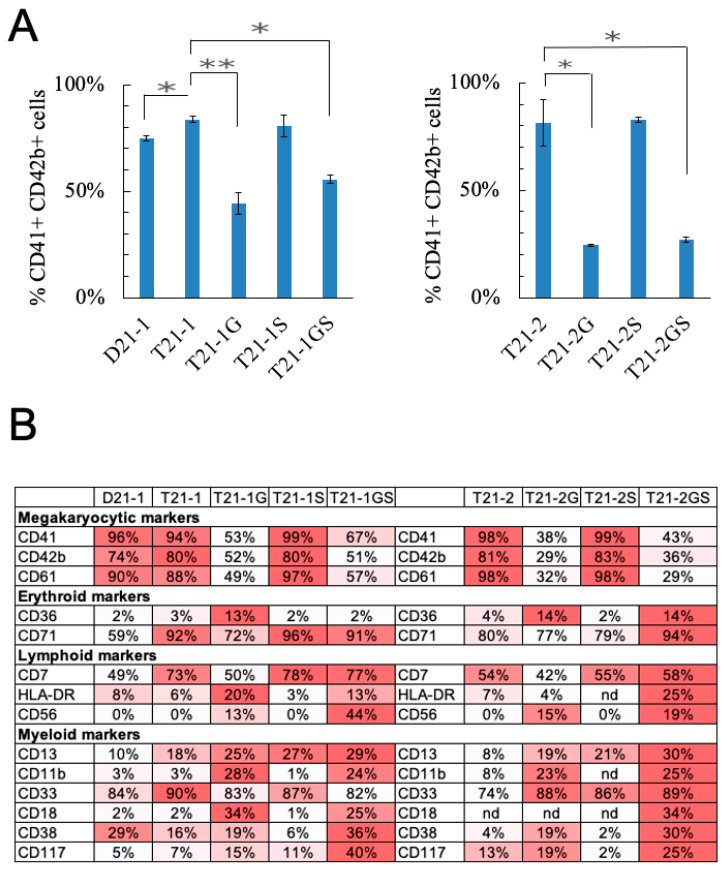
(**A**) Megakaryoblast population (CD41+CD42b+) in HSPCs cultured in media that promotes megakaryocytic lineage expansion is plotted. Error bars denote SE of the mean. Asterisk indicates statistical significance in indicated pair of iPSC lines, * *p* < 0.05, ** *p* < 0.01. (**B**) Immunophenotyping analysis of day 5 megakaryocytes. nd = not determined. The percentages of marker positive population are indicated. The darker color represents higher percentage.

Because the *STAG2* knockout co-operated with GATA1s for increasing megakaryoid population, we further evaluated the effect of *STAG2* knockout by culturing the day 10 HSPCs in megakaryocytic lineage-specific expansion media. The percentage of CD41+CD42b+ cells representing the mature megakaryocytes was significantly higher in T21-1 (83.7 ± 1.5%) compared to D21-1 (74.9 ± 1.3%) (Figure 3A, *p* = 0.019), in agreement with the previously reported effects of trisomy 21 on the promotion of erythro-megakaryocytic lineage expansion [27]. *GATA1* mutation in T21-1G reduced the percentage of megakaryocytes by 48.5% compared to T21-1 (*p* = 0.002). Similarly, T21-2G showed 57% decreased megakaryocytic population compared to T21-2 (*p* = 0.034). These results are consistent with the known effect of *GATA1* on megakaryocytic maturation [28]. *STAG2* mutation did not significantly alter the percentage of megakaryocytes in T21-1S and T21-2S compared to T21-1 and T21-2, respectively. The co-presence of *GATA1* and *STAG2* mutations reduced the percentage of CD41+CD42b+ population in T21-2GS (27.1 ± 1.2%) compared to T21-2 (81.5 ± 10.8%, *p* = 0.036). Similarly, the percent megakaryocytes in T21-1GS (55.5 ± 2.0%) were significantly lower than T21-1 (83.7 ± 1.5%, *p* = 0.007). There were no significant differences between the percent megakaryocytes in *GATA1 STAG2* mutants compared to *GATA1* mutants.

Methocult colony-forming assay was performed to further assess the multi-lineage colony-forming potential of the *GATA1* and *STAG2* mutant lines. Lines with *GATA1* mutation only formed CFU-GM colonies (Figure S2A), as we described previously [15]. Unlike *GATA1* mutation, *STAG2* mutation did not inhibit the formation of CFU-GEMM and BFU-E colonies (Figure S2B). CFU-GM colonies were lower, while CFU-GEMM colonies were higher in number in T21-2S compared to T21-2, although these differences were not statistically significant. The number of CFU-GM colonies in T21-1GS and T21-2GS was significantly reduced compared to T21-1G and T21-2G, respectively (Figure S2C, *p* = 0.010 and *p* = 0.002, respectively). These results indicate that the megakaryocyte maturation was hindered in *GATA1* and *GATA1 STAG2* mutant cells, while *STAG2* knockout alone did not have a significant effect.

### 3.6. Effect of STAG2 and GATA1 Mutations on the Immunophenotype of Megakaryocyte Lineage Expanded Cell Population

Immunophenotype analysis of the megakaryocyte lineage expanded cell population showed that the megakaryocytic markers CD41, CD42b, and CD61 were lower in *GATA1* mutated lines compared to their respective wildtype lines (Figure 3B). *STAG2* mutation alone did not alter the percentage of cells expressing these markers. Co-presence of *STAG2* mutation in GATA1s-expressing cells did not significantly alter the percentage of cells expressing megakaryocytic markers compared to *GATA1* mutant lines. 

Of note, the myeloid markers CD13 and CD11b are the only two markers expressed on the majority of DS-ML blasts in comparison with TAM blasts [29,30]. The percentage of CD13-expressing cells was higher in megakaryoblasts expanded from iPSC lines with *STAG2* knockout with or without *GATA1* mutation (Figure 3B). Interestingly, the CD13+ cell population was highest in T21-1GS (28.7%) and T21-2GS (29.7%) compared to any other line in the respective isogenic family. The percentage of CD11b+ cells was higher in the megakaryocytes with *GATA1* mutation, with or without *STAG2* mutation, but lower in wildtype or *STAG2* mutated cells. These data indicate that CD13 expression was triggered by *STAG2* knockout, and CD11b expression was promoted by GATA1s. These two mutations co-operatively enhanced DS-ML markers.

CD56, a marker with aberrant expression on TMD as well as DS-ML blasts [31,32], was expressed only in the megakaryoblasts expanded from iPSC lines with *GATA1* mutation, but not with *STAG2* knockout (Figure 3B). Furthermore, there was a statistically significant increase in CD56-expressing cells in the double-mutant line T21-1GS (43.8%, mean fluorescent intensity, MFI = 116,160 ± 2842) compared to T21-G1 (12.8%, *p* = 0.002; MFI = 21,262 ± 9077, *p* < 0.0001; Figure S3A,B). The CD56-expressing cell population was also higher in T21-2GS (19.0%, MFI = 41,910 ± 2924) compared to T21-2G (15.1%, MFI = 33,752 ± 937, *p* = 0.029).

STAG2 knockdown has been shown to promote stemness [33]. We tested the presence of stem cell markers on megakaryocytes generated by lineage expansion of HSPCs derived from iPSC lines with *GATA1* and/or *STAG2* mutation. CD117-expressing cells were highest in T21-1GS (*p* = 0.001) and T21-2GS (*p* = 0.048) lines when compared to their respective isogenic family of *GATA1* mutant lines (Figure 3B and Figure S3C). Neither single mutant of *GATA1* or *STAG2* showed a statistically significant increase in CD117+ cells compared to wildtype trisomic, suggesting that mutations in these two genes co-operate to increase the percentage of CD117+ cells representing stem cell-like population. A recent study highlighted the significance of CD117 in mediating leukemia progression in fetal liver HSC model [34].

Thus, GATA1s in collaboration with *STAG2* knockout altered the immunophenotype of the megakaryocytic population in favor of DS-ML and stem cell markers.

### 3.7. GATA1 Mutation with or without STAG2 Knockout Altered Distinct Signaling Pathways

**Figure 4:**
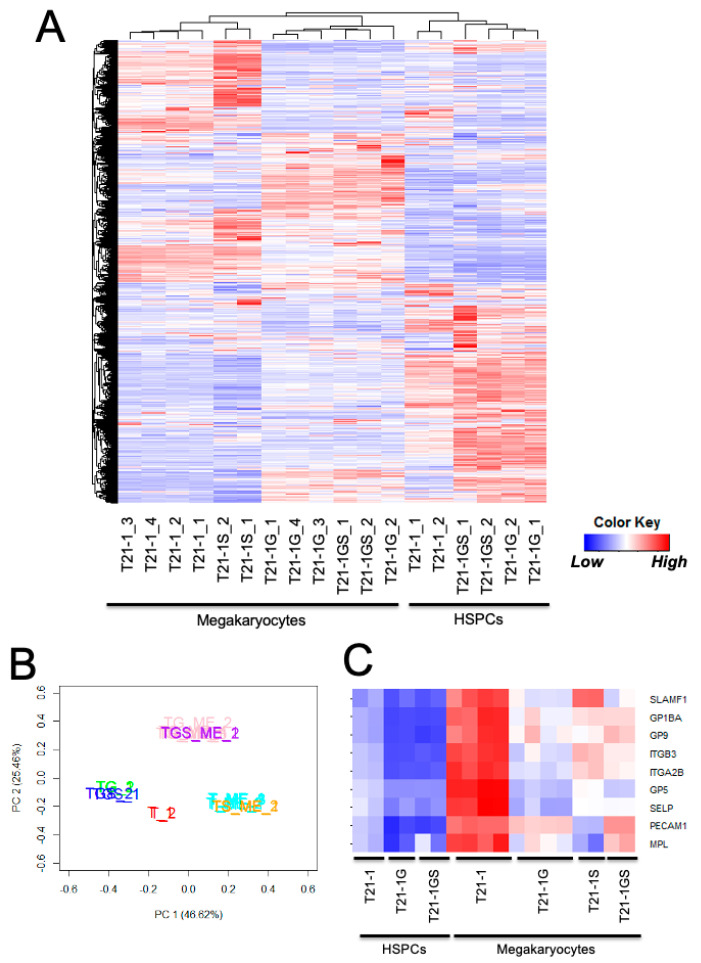
(**A**) Heatmap showing the unsupervised hierarchical clustering of all samples. (**B**) Principal component analysis of the indicated samples. T21-1 HSPCs (T_1,2 in red), T21-1G HSPCs (TG_1,2 in green), T21-1GS (TGS_1,2 in blue), T21-1 megakaryocytes (T_ME_1,2 in teal), T21-1G megakaryocytes (TG_ME_1,2 in pink), T21-1S (TS_ME_1,2 in yellow), T21-1GS (TGS_ME_1,2 in magenta). All genes were used for both analyses. (**C**) Heatmap showing the expression of megakaryocyte markers.

Transcriptome analysis of HSPCs (day 12 after hematopoietic differentiation) and megakaryocytes generated from the trisomic iPSCs with wildtype and mutants was conducted. A heatmap generated by unsupervised hierarchical clustering (FDR < 0.05) showed that the megakaryocyte samples clustered together, away from the HSPC samples, regardless of the mutation status (Figure 4A). Principal component analysis using all samples revealed four distinct groups (Figure 4B). The *GATA1* and *GATA1 STAG2* mutant HSPCs were very close to each other and away from wildtype HSPCs. The *STAG2* knockout megakaryocytes resided close to wildtype megakaryocytes, whereas the *GATA1* mutant megakaryocytes with or without *STAG2* mutation formed a separate cluster. These data indicate that the effect of GATA1s is dominant over *STAG2* knockout. 

In agreement with the immunophenotyping data (Figure 3B), megakaryocytic markers such as CD41 (*ITGA2B*), CD42b (*GP1BA*), and CD61 (*ITGB3*) were upregulated in wildtype megakaryocytes compared to HSPCs, but mutants had significantly lower expression compared to wildtype megakaryocytes (Figure 4C). Consistent with the prominent role of *GATA1* in megakaryocyte and erythrocyte differentiation [28,35], *GATA1* mutation had a strong suppressive effect on the genes belonging to the ‘platelet activation, signaling, and aggregation’ (Figure S4A) and ‘erythrocyte differentiation’ (Figure S4B) pathways. The C-MYB transcription factor pathway that negatively impacts megakaryocytic differentiation [35] was activated by *GATA1* mutation. *STAG2* knockout had minimal impact on C-MYB transcription factor network, and the expression profile of *GATA1 STAG2* double-mutant HSPCs was similar to *GATA1* mutant (Figure S5A). The upregulation of the C-MYB target, Bcl2, in *GATA1* and *GATA1 STAG2* mutant megakaryocytes compared to wildtype from both iPSC lines was confirmed at the protein level (Figure S5B).

**Figure 5:**
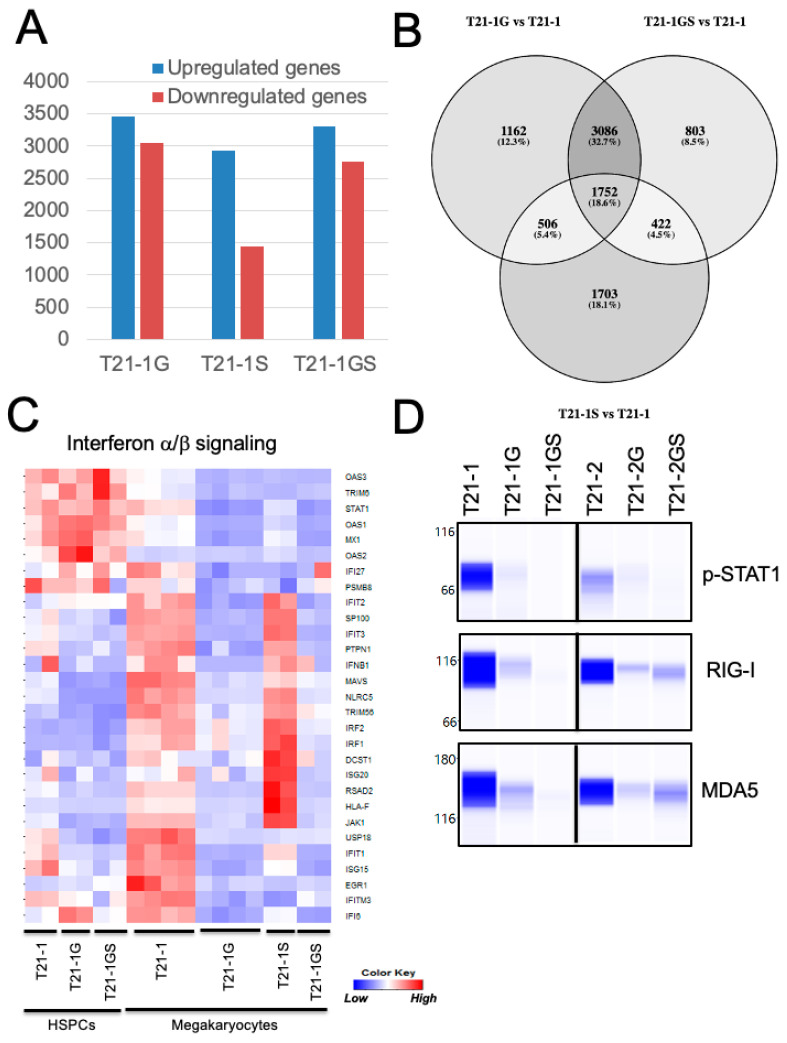
(**A**) Graph showing the number of differentially regulated genes (FDR < 0.05, log fold change >2 or <−2) in the indicated megakaryocytes with respect to wildtype T21-1. (**B**) Venn diagram of the differentially regulated genes. The shading is proportional to the percentage of shared genes. (**C**) Heat map showing the expression of genes belonging to the interferon α/β signaling pathway. (**D**) Wes immunoblot analysis of key proteins belonging to the interferon α/β signaling pathway in megakaryocytes. Total protein was quantitated using total protein analysis kit and used for normalization.

Genes differentially expressed in *GATA1* or *STAG2* single- and double-mutant megakaryocytes with respect to the wildtype megakaryocytes were listed (Table S1 and Figure S5A). There was maximal overlap (32.7%) in the genes differentially regulated in *GATA1* and *GATA1 STAG2* mutant megakaryocytes (Figure 5B). The top hits in the ‘biological processes’ and ‘pathways’ associated with differentially expressed genes were identified using Enrichr. The interferon α/β signaling pathway was identified as the topmost modulated pathway (Table S2) and biological processes (Table S3) in downregulated genes differentially expressed in both T21-1G and T21-1GS megakaryocytes. A gene set enrichment analysis also identified interferon α/β signaling pathway as significantly downregulated in *GATA1* mutant (Figure S6A). A heatmap of the genes belonging to this pathway showed that the interferon α/β signaling pathway was greatly suppressed in *GATA1* mutant but upregulated in *STAG2* knockout megakaryocytes compared to the wildtype (Figure 5C). The double mutant megakaryocytes also exhibited downregulation of this pathway. The retinoic acid-inducible gene-I (RIG-I) and the melanoma differentiation-associated protein 5 (MDA5) were identified as nodes that mediate the interferon signaling pathway [36,37] (Figure S6B). The reduction in RIG-I and MDA5 protein levels and the downregulation of phosphorylation of the interferon pathway effector STAT1 in megakaryocytes generated from both iPSC lines (Figure 5D) confirmed the inhibition of interferon pathway in *GATA1* mutant megakaryocytes, with or without *STAG2* mutation.

Genes upregulated in both T21-1G and T21-1GS megakaryocytes identified the toll-like receptor cascade, which is a pro-inflammatory pathway that initiates myeloid differentiation [38,39], and interleukin signaling, which functions downstream of the toll-like receptor cascade (Figure S6C and Table S2). While these pathways were activated by *GATA1* mutation, they were suppressed by *STAG2* knockout (Figure S6D), in agreement with previous reports of downregulation of inflammatory genes by STAG2 knockdown [40]. The co-presence of *STAG2* and *GATA1* mutation partially reverted the upregulation of these pathways, especially in megakaryocytes. We also observed an enrichment for myeloid/neutrophil-associated processes in ‘biological processes’ (Table S3) and an upregulation of several genes enriched in myeloid cells including ITGAM (*CD11b*), ANPEP (*CD13*), *CD14*, *ITGAX* (CD11c), *ADGRE1* (F4/80), *CD163*, *TREM2*, *MAFB*, and *SPI1* (PU.1) in *GATA1* mutants. The expression of these genes was also partially reverted in *GATA1 STAG2* mutant megakaryocytes (Figure S5C).

‘Extracellular matrix organization’ and ‘NCAM1 interaction’ pathways were also identified as two of the topmost modulated pathways (Table S2 and Figure S3B). *NCAM1* encodes CD56, which was not expressed in wildtype or *STAG2* knockout megakaryocytes but expressed at high levels in *GATA1* and *GATA1 STAG2* double-mutant megakaryocytes, as shown in the immunophenotype analysis described above (Figure S3A), thus validating the RNA-Seq data (Figure S3B). The genes belonging to the ‘extracellular matrix organization’ pathway were also upregulated in the double-mutant megakaryocytes. Taken together, *GATA1* and *STAG2* mutations had distinct effects on signaling pathways regulating megakaryocytic and myeloid differentiation.

## 4. Discussion

Trisomy 21 and GATA1s are necessary and sufficient for TAM [25]. However, these two events are not adequate for DS-ML leukemogenesis. Whole-genome and whole-exome sequencing analyses identified recurrent somatic mutations in DS-ML blasts, which were not detected in TAM blasts [11,12]. The association of these putative driver mutations was the basis of the general agreement in the field that acquisition of additional somatic mutations drives TAM to develop into DS-ML. In this study, using the iPSC model system, we provided experimental evidence showing that GATA1s and *STAG2* knockout in the trisomy 21 background co-operatively increased the percentage of megakaryoid population and promoted the expression of DS-ML and stem cell markers.

*GATA1* encodes a transcription factor that plays a prominent role in the proliferation and differentiation of hematopoietic lineage cells including erythrocytes and megakaryocytes [28,41]. Consistent with this role, *GATA1* mutation had a strong suppressive effect on the genes belonging to the ‘platelet activation, signaling, and aggregation’. STAG2 protein functions as a transcriptional coactivator that regulates chromatin accessibility and thereby transcription of hematopoietic lineage-specification genes *EBF1* and *PAX5* [26]. *STAG2* loss in murine HSPCs has been shown to increase self-renewal and reduce differentiation. In our study, *STAG2* knockout showed suppression of ‘platelet activation, signaling, and aggregation’ pathway, though not as prominent as *GATA1* mutation. Thus, the megakaryocyte differentiation was severely hampered in the double-mutant HSPCs, resulting in the observed co-operative increase in the percentage of immature megakaryocytic (megakaryoid) population.

Although immunophenotypic differences between TAM and DS-ML blasts are not prominent, a small number of studies attempting to tease out these differences showed increased presence of CD13 and CD11b in DS-ML blasts compared to TAM blasts [29,30]. *STAG2* knockout in the presence of GATA1s and trisomy 21 increased CD13 and CD11b-expressing population representing a DS-ML-like immunophenotype. Studies are in progress in the laboratory to evaluate the in vivo engraftment potential of the double-mutant HSPCs in comparison with single-mutant cells.

A CRISPR/Cas9 screen in disomic background showed that the loss of some of the recurrently mutated DS-ML genes in a murine model of GATA1s overexpression led to the aberrant proliferation of lineage cell types [42]. However, mutations in cohesin complex components (especially *STAG2*), which constitute a major subtype of DS-ML recurrent mutations, were not found by this screen. The results suggest that it is likely that the first genetic event (i.e., trisomy 21) necessary for providing the cellular context for acquisition of additional somatic mutations was necessary to manifest the synergism between *STAG2* mutation and GATA1s. Recently, it was shown that preleukemia to leukemia transition in fetal liver trisomic HSCs was mediated by mutations in cohesion genes in addition to *GATA1* [34]. Our results are consistent with this study, suggesting that hematopoietic differentiation of gene targeted iPSCs is a suitable model for rare leukemia subtypes.

The downregulation of the interferon α/β signaling pathway, observed in mice engineered to produce GATA1s [43], has been considered as one of the potential events leading to leukemogenesis in DS-ML [44]. Our identification of this pathway in genes differentially regulated in *GATA1 STAG2* double-mutant megakaryocytes supports the notion that DS-ML-like characteristics are acquired by the co-operation between these two mutations. *GATA1* mutation triggered pathways such as toll-like receptor signaling that promote myeloid differentiation. *GATA1 STAG2* double mutation countered the expression of genes affecting myeloid differentiation. These data are consistent with the increased preponderance of myeloid population in *GATA1* mutant HSPCs and suppression of myeloid population in *STAG2* knockout HSPCs. Thus, GATA1s-mediated activation of toll-like receptor signaling likely leads to myeloid commitment, while the *STAG2* knockout halts myeloid differentiation and promotes self-renewal leading to leukemia. In addition, we identified ‘extracellular matrix organization’ and ‘NCAM1 interactions’ to be upregulated in the double mutant megakaryocytes. The role of the extracellular matrix in modulating the bone marrow microenvironment to favor leukemogenesis is well known [45]. Specifically, NCAM1 interactions have been shown to be involved in the maintenance of leukemic stem cells [46]. Thus, the upregulation of these pathways in *GATA1 STAG2* mutant megakaryocytes alongside downregulation of interferon α/β signaling pathway is likely responsible for the transformation to the DS-ML phenotype. In summary, we identified specific signaling pathways by which co-operative mutations in *GATA1* and *STAG2* may develop leukemia in a trisomy background.

In addition to *STAG2,* representing cohesion complex components, mutations in genes encoding epigenetic regulators, and signaling molecules [10,11,12] have also been associated with DS-ML leukemogenesis. It is interesting to study the impact of each of these co-occurring mutations in the presence of trisomy 21 and GATA1s on megakaryoid and myeloid development leading to leukemogenesis. It is likely that although distinct, each of these co-occurring mutations may alter the GATA1s-binding sites and/or the epigenomic landscape, resulting in gene expression that favors malignant transformation. The development of such additional models is currently in progress in our laboratory.

## 5. Conclusions

GATA1s and *STAG2* knockout co-operatively increased the megakaryoid population and enhanced expression of DS-ML and stem cell markers, closely resembling the DS-ML immunophenotype. These two mutations induced specific signaling pathways that halted megakaryocytic differentiation and promoted self-renewal. Thus, using CRISPR/Cas9 gene editing of trisomy 21 iPSCs, we provide experimental evidence for the co-operation between trisomy 21, GATA1s, and *STAG2* knockout.

## Data Availability

Data presented in this article are available at GSE188568 and in the Supplementary Material.

## Supplementary Materials

The following are available online at https://www.mdpi.com/article/10.3390/cells11040628/s1, Figures S1–S7. Figure S1: Percentage of erythroid, megakaryoid and myeloid cells in HSPCs generated by hem-atopoietic differentiation of indicated iPSC lines with or without GATA1 and/or STAG2 mutation; Figure S2: (A) Representative images of CFU-GEMM, CFU-GM and BFU-E colonies in a Methocult colony-forming assay. (B) The average number of CFU-GEMM, CFU-GM and BFU-E colonies from 3–5 independent experiments were plotted; Figure S3: (A) CD56 cell surface expression in megakaryocytes generated from hematopoietic differentiation of iPSCs. Average data from three independent experiments was plotted. (B) Heatmap showing NCAM1 expression. (C) CD117 cell surface expression in megakaryocytes generated from hematopoietic differentiation of iPSCs; Figure S4: Heatmaps showing the expression of genes belonging to ‘platelet activation, signaling and aggregation’ pathway; Figure S5: (A) Heat map showing the expression of genes belonging to the C-MYB transcription factor network. (B) Wes immunoblot analysis of key protein belonging to the C-MYB transcription factor network in megakaryocytes. (C) Heatmap showing the expression of myeloid markers; Figure S6: (A) Gene set enrichment analysis showing suppressed type I interferon response in GATA1 mutant megakaryocytes. (B) Heat map showing the expression of genes be-longing to the RIG-I/MDA5 mediated induction of interferon α/β signaling pathway. (C) Gene set enrichment analysis showing increased toll-like receptors signaling in GATA1 mutant megakar-yocytes. (D) Heat maps showing the expression of genes belonging to the Toll-like receptors cascade and interleukin signaling; Figure S7: Heatmap showing the expression of genes belonging to ‘ex-tracellular matrix organization’ pathway. Table S1. List of differentially regulated genes in indicated mutant megakaryocytes compared to wildtype. Table S2. List of pathways identified by Enrichr analysis. Table S3. List of ‘biological processes’ identified by Enrichr analysis.
