# Supplementary material for: Modeling Down Syndrome Myeloid Leukemia by Sequential Introduction of GATA1 and STAG2 Mutations in Induced Pluripotent Stem Cells with Trisomy 21
Source: Cells. 2022 Feb 11;11(4):628. doi: 10.3390/cells11040628 (PMC8870267; doi:10.3390/cells11040628)
Supplement: Supplementary file 1 [file cells-11-00628-s001.zip › Supplementary_new.pdf]

Modeling Down syndrome myeloid leukemia by CRISPR/Cas9 mediated introduction of *STAG2* mutation in *GATA1* mutated trisomic induced pluripotent stem cells

Sonali P. Barwe[1], Aimy Sebastian[2], Ishnoor Sidhu[1], E. Anders Kolb[1], Anilkumar Gopalakrishnapillai[1] *

[1]Nemours Centers for Childhood Cancer Research and Cancer & Blood Disorders, Nemours Children's Hospital, Wilmington, DE 19803

[2]Lawrence Livermore National Laboratory, Livermore, CA 94550

*Address of correspondence:

Anilkumar Gopalakrishnapillai

Phone: (302) 651-4833

Fax: (302) 651-4827

Email: anil.g@nemours.org

Keywords: Down syndrome, iPSC, CRISPR/Cas9, leukemia, GATA1s, *STAG2*

Running title: Modeling DS-ML using iPSCs and CRISPR/Cas9

Disclosures: The authors declare no potential conflicts of interest.

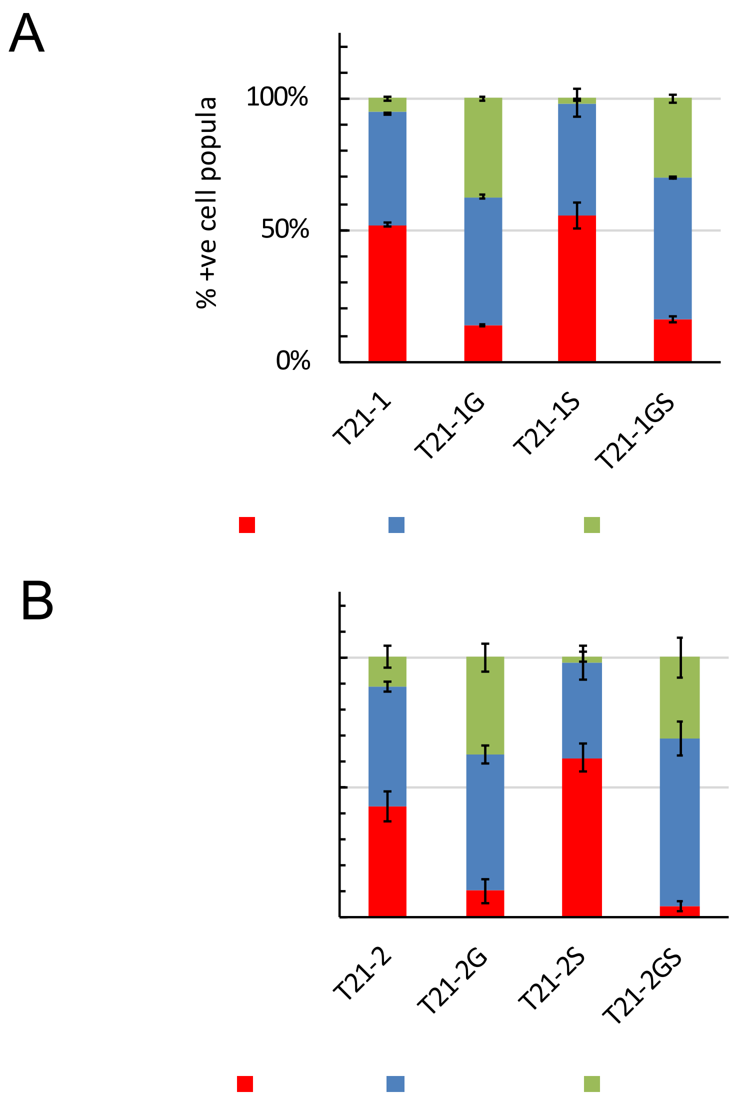

Supplementary Figure. S1 A, B) Percentage of erythroid, megakaryoid and myeloid cells in HSPCs generated by hematopoietic differentiation of indicated iPSC lines with or without *GATA1* and/or *STAG2* mutation.

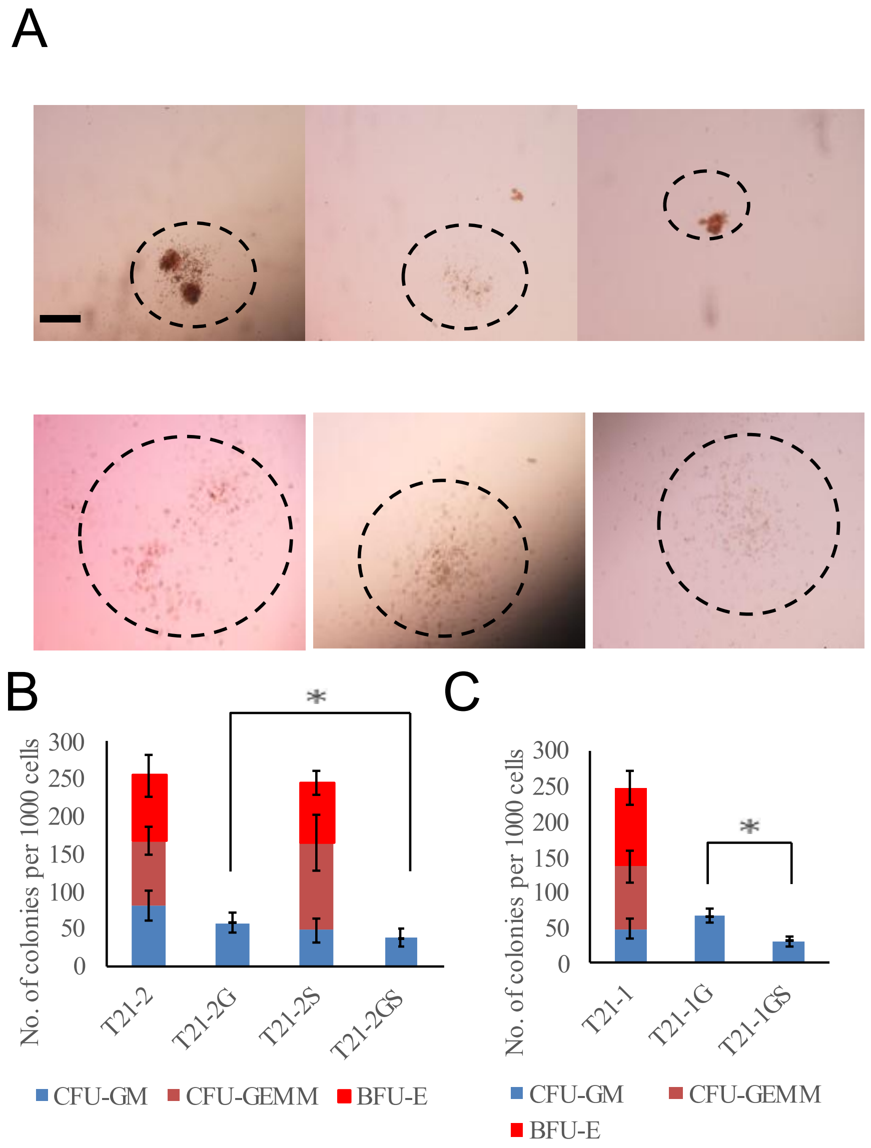

Supplementary Figure. S2 A) Representative images of CFU-GEMM, CFU-GM and BFU-E colonies in a Methocult colony-forming assay. (**B)** The average number of CFU-GEMM, CFU-GM and BFU-E colonies from 3-5 independent experiments were plotted. Error bars indicate SD of the Mean. Asterisk denotes statistical significance ($P<0.05$).

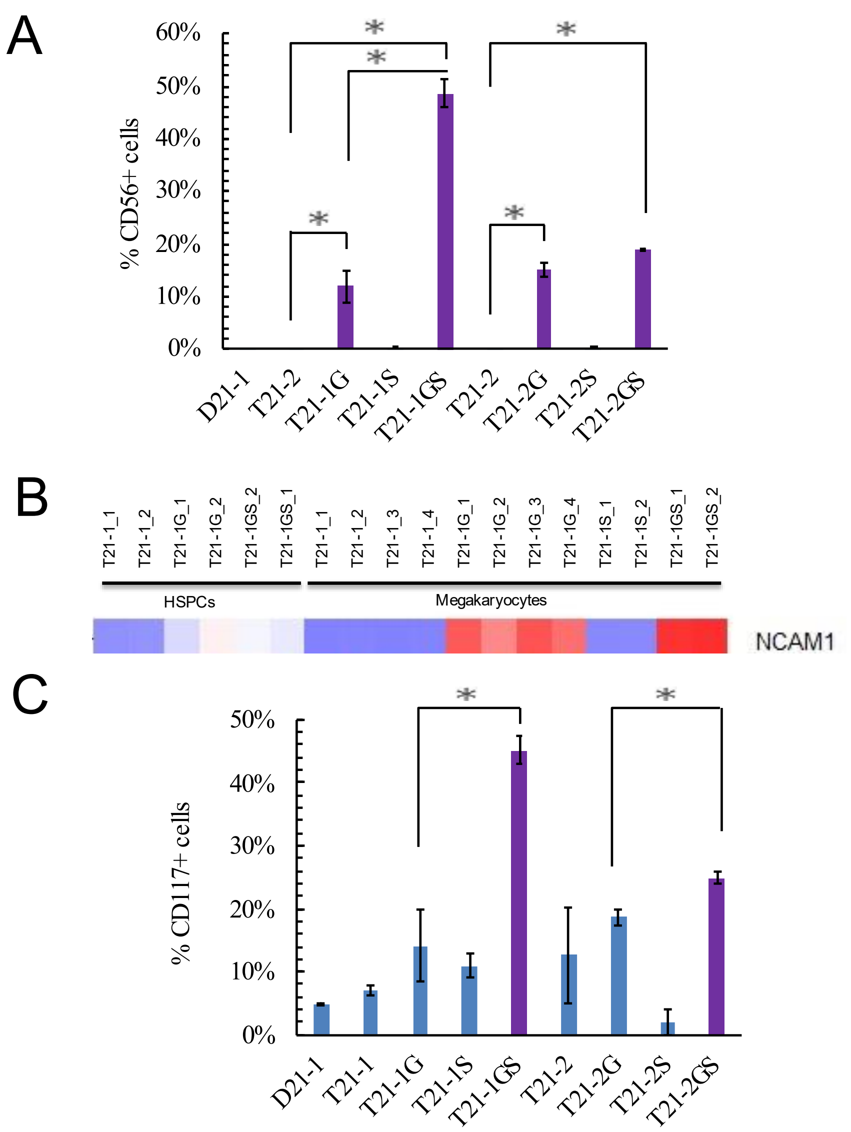

Supplementary Figure. S3 (**A**) CD56 cell surface expression in megakaryocytes generated from hematopoietic differentiation of iPSCs. Average data from three independent experiments was plotted. *P <0.05. (**B**) Heatmap showing NCAM1 expression. (**C**) CD117 cell surface expression in megakaryocytes generated from hematopoietic differentiation of iPSCs. Average data from three independent experiments was plotted. *P <0.05.

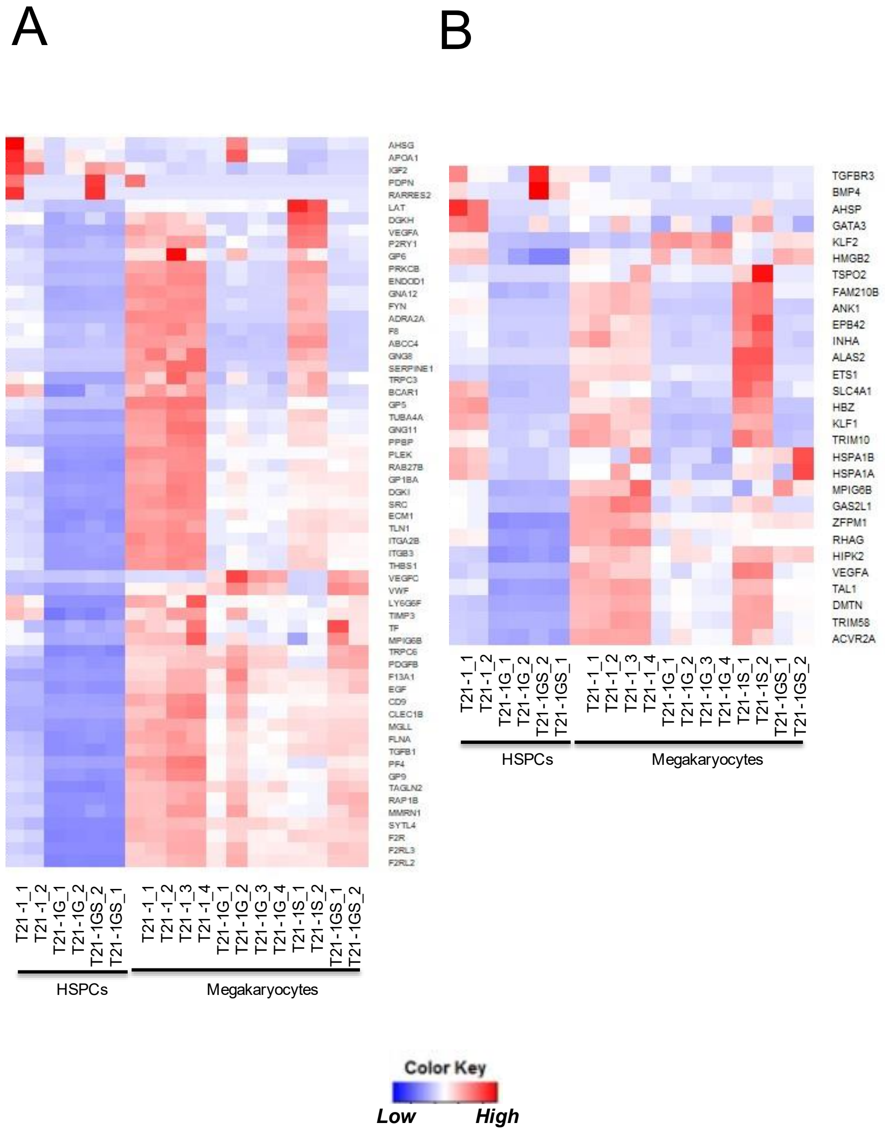

Supplementary Figure. S4 Heatmaps showing the expression of genes belonging to ‘platelet activation, signaling and aggregation’ pathway (**A**) and ‘erythrocyte differentiation’ pathway (**B**).

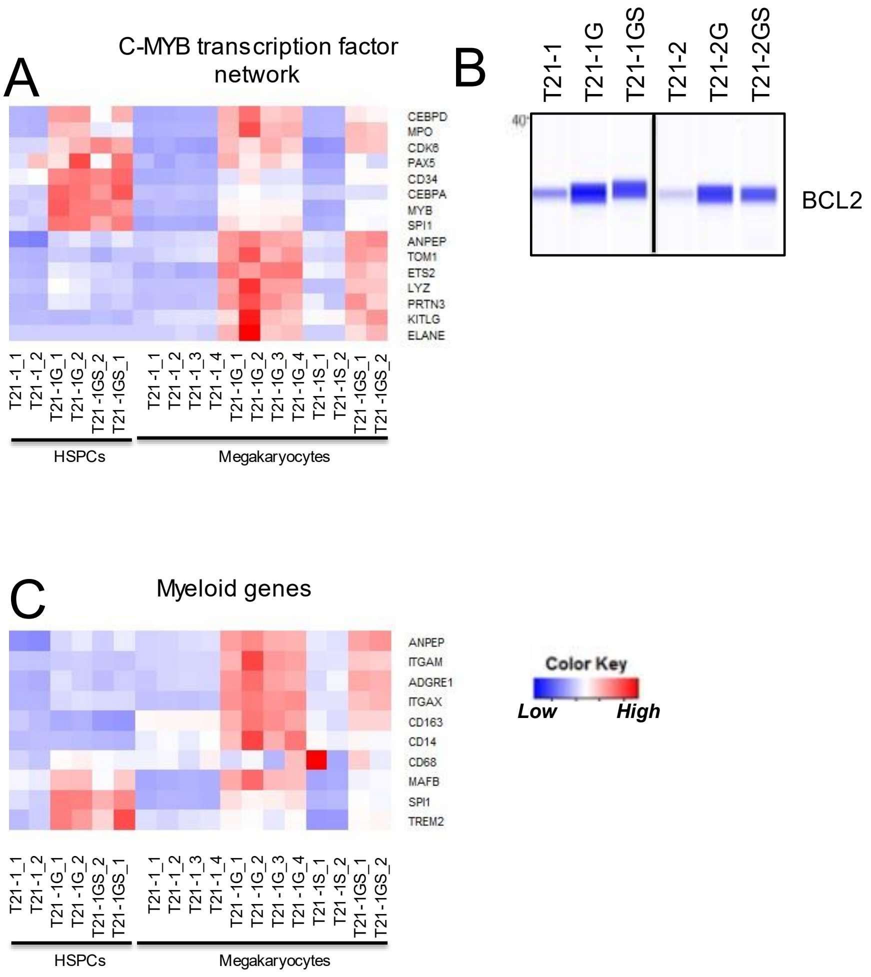

Supplementary Figure. S5 A) Heat map showing the expression of genes belonging to the C-MYB transcription factor network. **B)** Wes immunoblot analysis of key protein belonging to the C-MYB transcription factor network in megakaryocytes. Total protein was quantitated using total protein analysis kit and used for normalization. **C**) Heatmap showing the expression of myeloid markers.

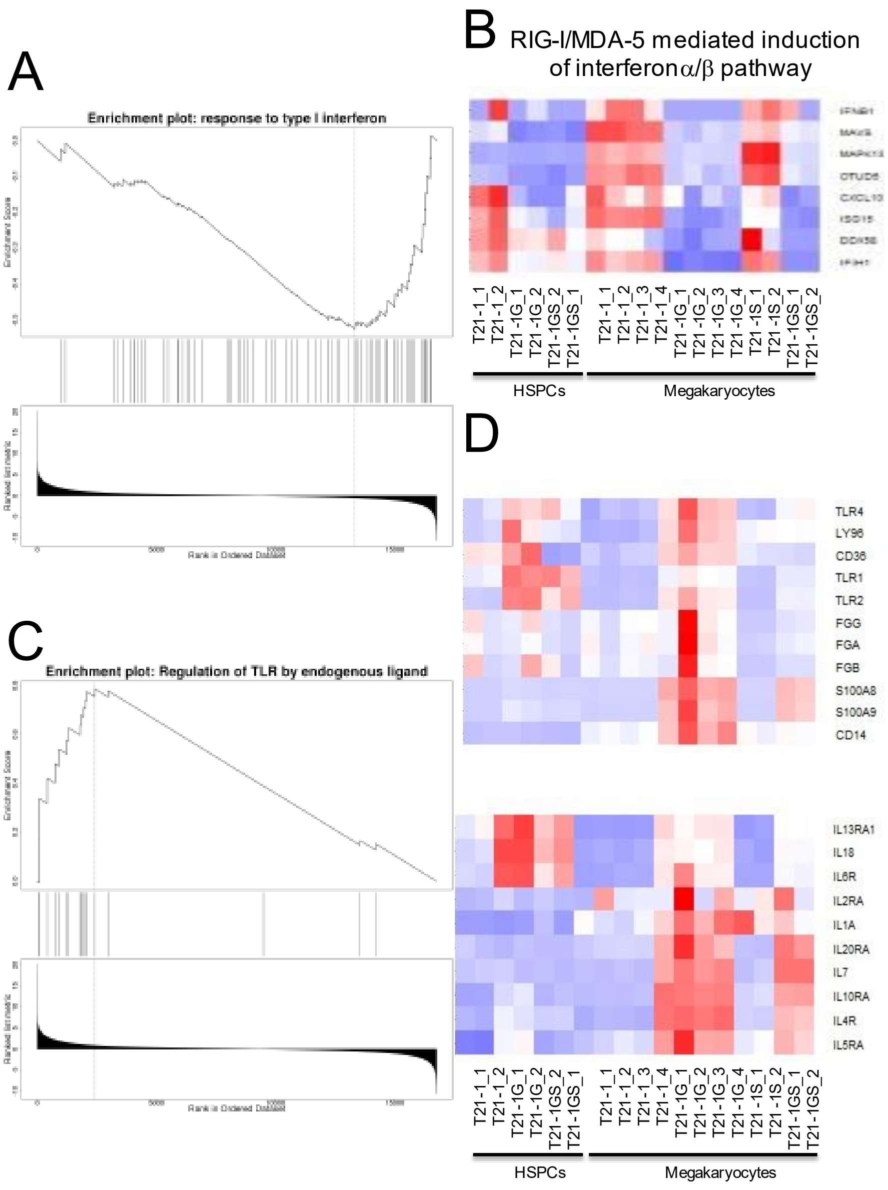

Supplementary Figure. S6 A) Gene set enrichment analysis showing suppressed type I interferon response in *GATA1* mutant megakaryocytes. **B)** Heat map showing the expression of genes belonging to the RIG-I/MDA5 mediated induction of interferon β/α signaling pathway. **C)** Gene set enrichment analysis showing increased toll-like receptors signaling in *GATA1* mutant megakaryocytes. **D)** Heat maps showing the expression of genes belonging to the Toll-like receptors cascade and interleukin signaling.

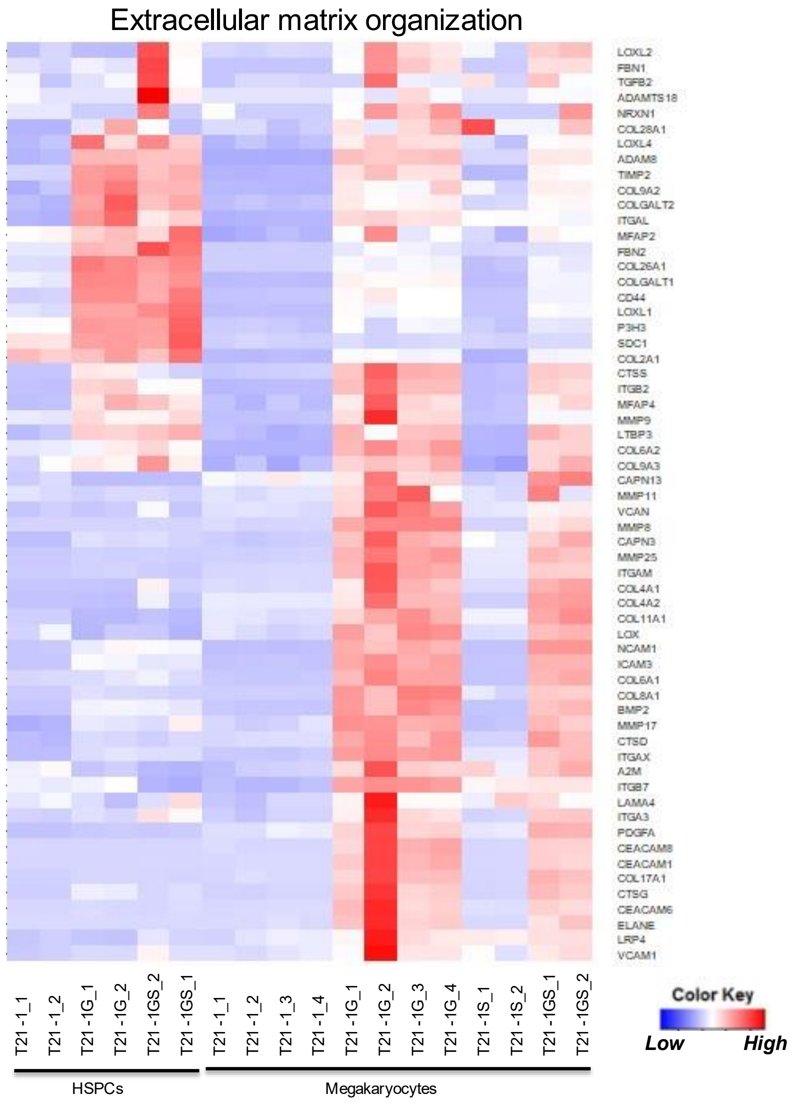

Supplementary Figure. S7 Heatmap showing the expression of genes belonging to 'extracellular matrix organization' pathway.

Supplementary Table S1 List of differentially regulated genes in indicated mutant megakaryocytes compared to wild type.

Supplementary Table S2 List of pathways identified by Enrichr analysis.

Supplementary Table S3 List of 'biological processes' identified by Enrichr analysis
